# Use of Self-Collected Dried Blood Spots and a Multiplex Microsphere Immunoassay to Measure IgG Antibody Response to COVID-19 Vaccines

**DOI:** 10.1128/spectrum.01336-22

**Published:** 2023-01-09

**Authors:** Katherine L. Nemeth, Erica Yauney, Jean M. Rock, Rachel Bievenue, Monica M. Parker, Linda M. Styer

**Affiliations:** a Wadsworth Center, New York State Department of Health, Albany, New York, USA; b Department of Biomedical Sciences, School of Public Health, University at Albany, Albany, New York, USA; University of Mississippi Medical Center

**Keywords:** dried blood spots, SARS-CoV-2, self-collected, vaccine, immunoassays, serosurveys

## Abstract

Serosurveys can determine the extent and spread of a pathogen in populations. However, collection of venous blood requires trained medical staff. Dried blood spots (DBS) are a suitable alternative because they can be self-collected and stored/shipped at room temperature. As COVID-19 vaccine deployment began in early 2021, we rapidly enrolled laboratory employees in a study to evaluate IgG antibody levels following vaccination. Participants received a DBS collection kit, self-collection instructions, and a brief questionnaire. Three DBS were collected by each of 168 participants pre- and/or postvaccination and tested with a multiplex microsphere immunoassay (MIA) that separately measures IgG antibodies to SARS-CoV-2 spike-S1 and nucleocapsid antigens. Most DBS (99.6%, 507/509) were suitable for testing. Participants with prior SARS-CoV-2 infection (*n* = 7) generated high S antibody levels after the first vaccine dose. Naïve individuals (*n* = 161) attained high S antibody levels after the second dose. Similar antibody levels were seen among those vaccinated with Moderna (*n* = 29) and Pfizer-BioNTech (*n* = 137). For those receiving either mRNA vaccine, local side effects were more common after the first vaccine dose, whereas systemic side effects were more common after the second dose. Individuals with the highest antibody levels in the week prior to the second vaccine dose experienced more side effects from the second dose. Our study demonstrated that combining self-collected DBS and a multiplex MIA is a convenient and effective way to assess antibody levels to vaccination and could easily be used for population serosurveys of SARS-CoV-2 or other emerging pathogens.

**IMPORTANCE** Serosurveys are an essential tool for assessing immunity in a population (1, 2). However, common barriers to effective serosurveys, particularly during a pandemic, include high-costs, resources required to collect venous blood samples, lack of trained laboratory technicians, and time required to perform the assay. By utilizing self-collected dried blood spots (DBS) and our previously developed high-throughput microsphere immunoassay, we were able to significantly reduce many of these common challenges. Participants were asked to self-collect three DBS before and/or after they received their COVID-19 vaccines to measure antibody levels following vaccination. Participants successfully collected 507 DBS that were tested for IgG antibodies to the spike and nucleocapsid proteins of SARS-CoV-2. When used with self-collected DBS, our relatively low-cost assay significantly reduced common barriers to collecting serological data from a population and was able to effectively assess antibody response to vaccination.

## INTRODUCTION

Serosurveys can collect information necessary to understand and respond to the spread of infectious diseases in a population. Serosurveys can also provide information regarding antibody prevalence, antibody waning, and susceptibility of populations with low seroprevalence to future outbreaks (1, 2). Early in the SARS-CoV-2 pandemic, our laboratory developed a high-throughput microsphere immunoassay (MIA) to detect IgG antibodies to the SARS-CoV-2 nucleocapsid and spike S1 antigens in dried blood spots (DBS). In April 2020, we used this assay to test >15,000 DBS collected by trained staff at grocery stores throughout New York State (NYS) and reported an estimated cumulative incidence of SARS-CoV-2 infection of 22.7% in New York City and 14% statewide (3). This assay was then employed for additional NYS serosurveys through June 2020 (4) and a large-scale serosurvey of DBS collected from all newborns born in NYS from November 2019 through November 2021 (5).

With the introduction of COVID-19 vaccines, we realized a need for DBS collected from individuals before and after vaccination to evaluate the ability of our MIA to measure antibody levels after vaccination. The first two COVID-19 vaccines, Pfizer-BioNTech and Moderna, received U.S. Food and Drug Administration Emergency Use Authorization (EUA) in December 2020 (6, 7), and a third vaccine, Janssen, was authorized in February 2021 (8). All three vaccines induce antibodies to the SARS-CoV-2 spike antigen. The Pfizer-BioNTech and Moderna vaccines are both mRNA-based vaccines while the Janssen vaccine is an adenovirus vector vaccine. In order to rapidly deploy our study without burdening health care staff, we chose to use self-collected DBS, a technique which has previously been used successfully for serosurveys (9–11). Therefore, we developed a DBS self-collection kit, instructions, and a brief questionnaire to collect demographic and COVID-19 history data. Wadsworth Center laboratory employees were recruited to self-collect DBS before, when possible, and after COVID-19 vaccination. DBS were tested using our multiplex MIA to measure antibody levels following vaccination in our study population.

## RESULTS

A total of 175 individuals were enrolled in this study. Four participants did not submit any samples, three submitted only one sample, and 168 (96%) submitted a full series of three DBS. A total of 509 DBS collected either at the participant’s home or at the worksite were received from 171 individuals. Two samples were deemed unsuitable for testing because they were received more than 14 days after being collected, leaving 507 (99.6%) suitable for testing. Individuals reported having some difficulty collecting a sample for 71 (14.0%) of the 509 specimens received. Most of these issues (11.8%) were associated with lack of blood flow or fast clotting and were commonly resolved by pricking a different finger, exercising, or taking a warm shower before collecting the sample. Other reported issues included messy collection (1.2%), difficulty working lancets (<1%), and not following instructions properly (<1%). Other issues related to not following instructions included DBS cards that were not fully dry upon receipt in the laboratory (1.4%), overlapping drops of blood (1.2%), missing paperwork (<1%), and names written on cards which should have only been identifiable by barcodes (<1%). None of these issues prevented the samples from being tested.

### Participant characteristics.

Participant ages ranged from 20 to over 65 years old with most participants falling between 40 and 59 years of age (Table 1). Most of the participants were female, White, and not Hispanic or Latino. Most participants lived in NYS with the majority residing in Albany County and the surrounding counties of Rensselaer, Saratoga, and Schenectady. All three FDA-authorized or approved vaccines are represented in our sample (Pfizer-BioNTech, Moderna, and Janssen). Most participants received the Pfizer-BioNTech vaccine while only three individuals received Janssen. Individuals received their first vaccine dose between December 23, 2020 and May 7, 2021 with a median date of March 4, 2021.

**TABLE 1:** Characteristics of the study participants^*a*^

Characteristics	#	%
Total enrolled	175	
No samples submitted	4	
Complete series submitted	168	
Age group (yrs)		
20 to 29	19	10.9
30 to 39	38	21.7
40 to 49	39	22.3
50 to 59	49	28.0
60+	30	17.1
Sex		
Female	122	69.7
Male	53	30.3
Race		
Asian	7	4.1
Black or African American	5	2.9
White	157	91.8
Other/Unknown	2	1.2
Ethnicity		
Hispanic or Latino	4	2.3
Not Hispanic or Latino	166	97.1
No response	1	0.6
Vaccine manufacturer		
Pfizer-BioNTech	137	80.1
Moderna	29	17.0
Janssen	3	1.8
County of Residence		
Albany	88	51.5
Rensselaer	27	15.8
Saratoga	26	15.2
Schenectady	14	8.2
Greene	4	2.3
Columbia	3	1.8
Other	9	5.3

a Age and sex were collected at time of enrollment and are reported for all enrolled individuals (*n* = 175). Race, ethnicity, and county were collected with first sample submission and are reported for individuals who submitted at least one sample (*n* = 171). Vaccine manufacturer was reported for all individuals who submitted at least one sample post 1st vaccine dose (*n* = 169).

Seven individuals in the study were infected with SARS-CoV-2 prior to beginning the study (4.1%) and two became infected during the study (1.1%). Of these nine individuals, six became infected in December 2020 while the others were infected in February and March 2021. All infected individuals received a mRNA vaccine. Eight of these individuals experienced COVID-19 symptoms and received a COVID-19 diagnosis (Table 2). One individual did not receive a COVID-19 diagnosis but had an initial nonreactive sample followed by a sample with IgG index values >1 for both N bead sets and was therefore classified as having been infected. For this study, recovered individuals are defined as those who were infected with COVID-19 prior to beginning the study. Naïve individuals are defined as individuals who were not infected prior to beginning the study, including the two individuals who became infected during the study. All three COVID-19 vaccines in this study induced spike (S) antibodies while natural infection with SARS-CoV-2 typically induced both nucleocapsid (N) and S antibodies in immunocompetent individuals. At their initial sample, recovered individuals had an IgG index value between 0.34 and 12.18 (mean index value = 3.61) for the N-SB bead set and between 1.48 and 5.84 (mean index value = 3.10) for the N-NA bead set. Prevaccine samples from naive individuals had IgG index values between 0.02 and 3.22 (mean index value = 0.19) for the N-SB bead set and between 0.04 and 0.91 (mean index value = 0.28) for the N-NA bead set. The one individual with a reactive result for N-SB (index value = 3.22) had nonreactive results for N-NA (index value = 0.79) and S (index value = 0.04) and thus was classified as naive.

**TABLE 2:** Summary of COVID-19 infected individuals

Characteristics	#	%
Infection status^*a*^		
Previous infection	7	
Concurrent infection	2	
COVID-19 diagnosis		
Yes	8	89%
No	1	11%
Positive PCR test		
Yes	6	67%
No	3	33%
COVID-19 symptoms		
Yes	8	89%
No	1	11%
Hospitalization		
Yes	0	0%
No	9	100%
Sex		
Female	6	67%
Male	3	33%
Age group (yrs)		
20 to 29	1	11%
30 to 39	4	44%
40 to 49	1	11%
50 to 59	3	33%
Race		
White	9	100%
Ethnicity		
Not Hispanic	9	100%

a Positive infection status was determined by report of a PCR positive test or a nucleocapsid index value greater than 1.

### Effect of previous infection on IgG S antibody levels.

**FIG 1:**
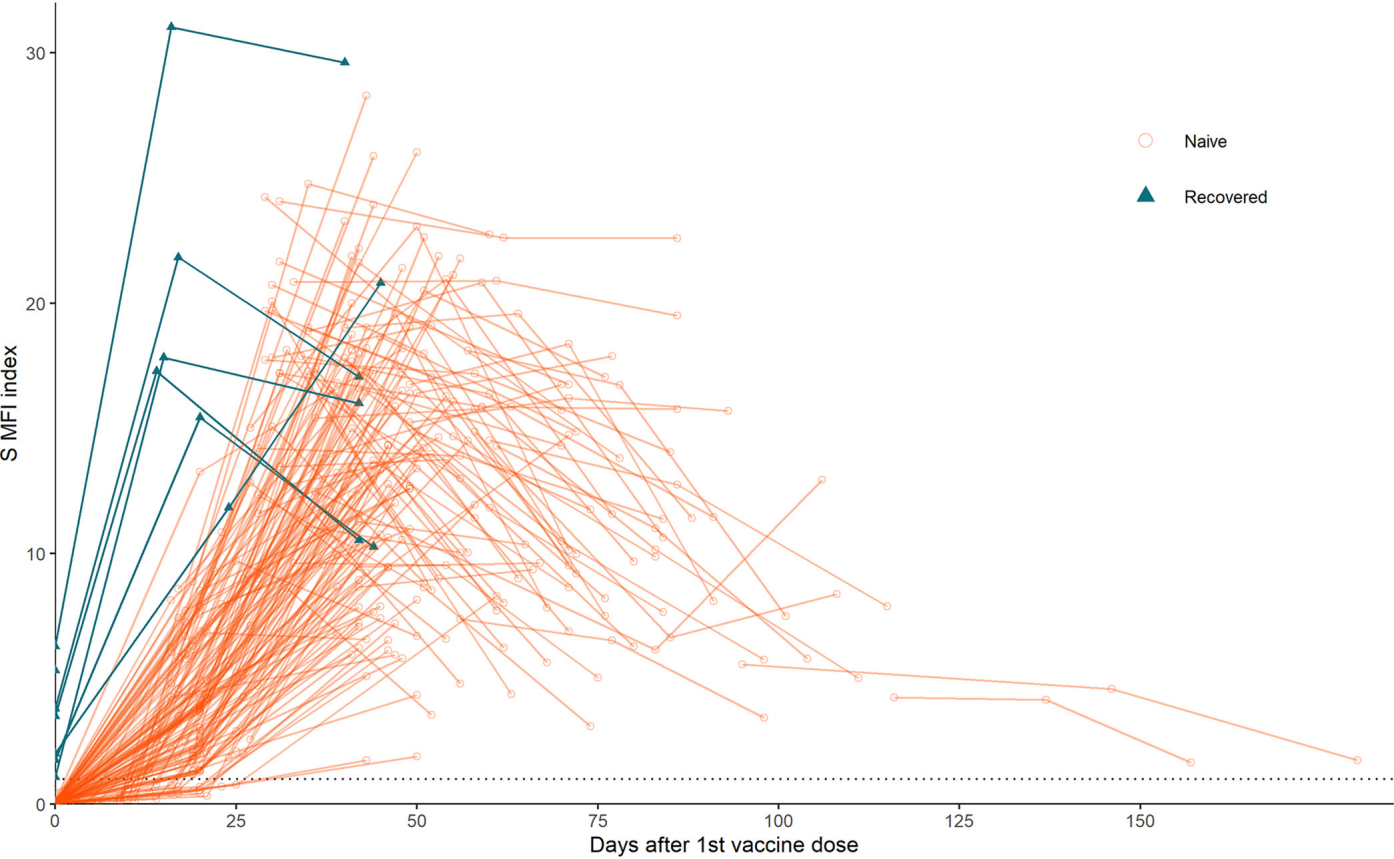
Individual SARS-CoV-2 spike IgG antibody responses to vaccination by infection status. Figure includes individuals vaccinated with Pfizer-BioNTech, Moderna, and Janssen vaccines. Recovered individuals includes all individuals infected prior to the beginning of the study. Dotted line indicates reactive cutoff.

All naive individuals were nonreactive for IgG S antibodies prior to receiving a vaccine dose (mean index = 0.05; range 0.01 to 0.49) while all recovered individuals were reactive (mean index = 3.40; range 1.07 to 6.29, *P* < 0.01) (Fig. 1). Recovered individuals showed an abrupt increase in S antibody levels after their first dose compared to naive individuals who showed a gradual increase between their first and second dose (15 to 21 days post dose 1, naive mean index = 3.90, recovered mean index = 21.50, U = 0, *P* = 0.0008). Naïve individuals’ S antibody levels increased rapidly after a second dose of either mRNA vaccine to reach levels similar to those of the recovered individuals (naive versus recovered:36 to 42 days post dose 1, U = 68 *P* = 0.28; 42 to 49 days post dose 1, U = 42.5, *P* = 0.65). S antibody levels in participants peaked several weeks after the second dose of either mRNA vaccine before gradually declining.

Recovered individuals submitted their first DBS prior to vaccination and between 60 and 120 days post symptom onset. In order to compare antibody levels induced by mRNA vaccines to those induced by natural infection, the initial samples submitted by all recovered individuals were compared to naive individuals who submitted samples between 60 and 120 days after their second vaccine dose. The S index values of recovered individuals induced by natural infection alone were significantly lower than S index values induced by vaccination in naive individuals in the same time frame (recovered mean = 3.40, naive mean = 9.53, U = 159, *P* = 0.0004).

### Effect of vaccine manufacturer on IgG S antibody levels.

**FIG 2:**
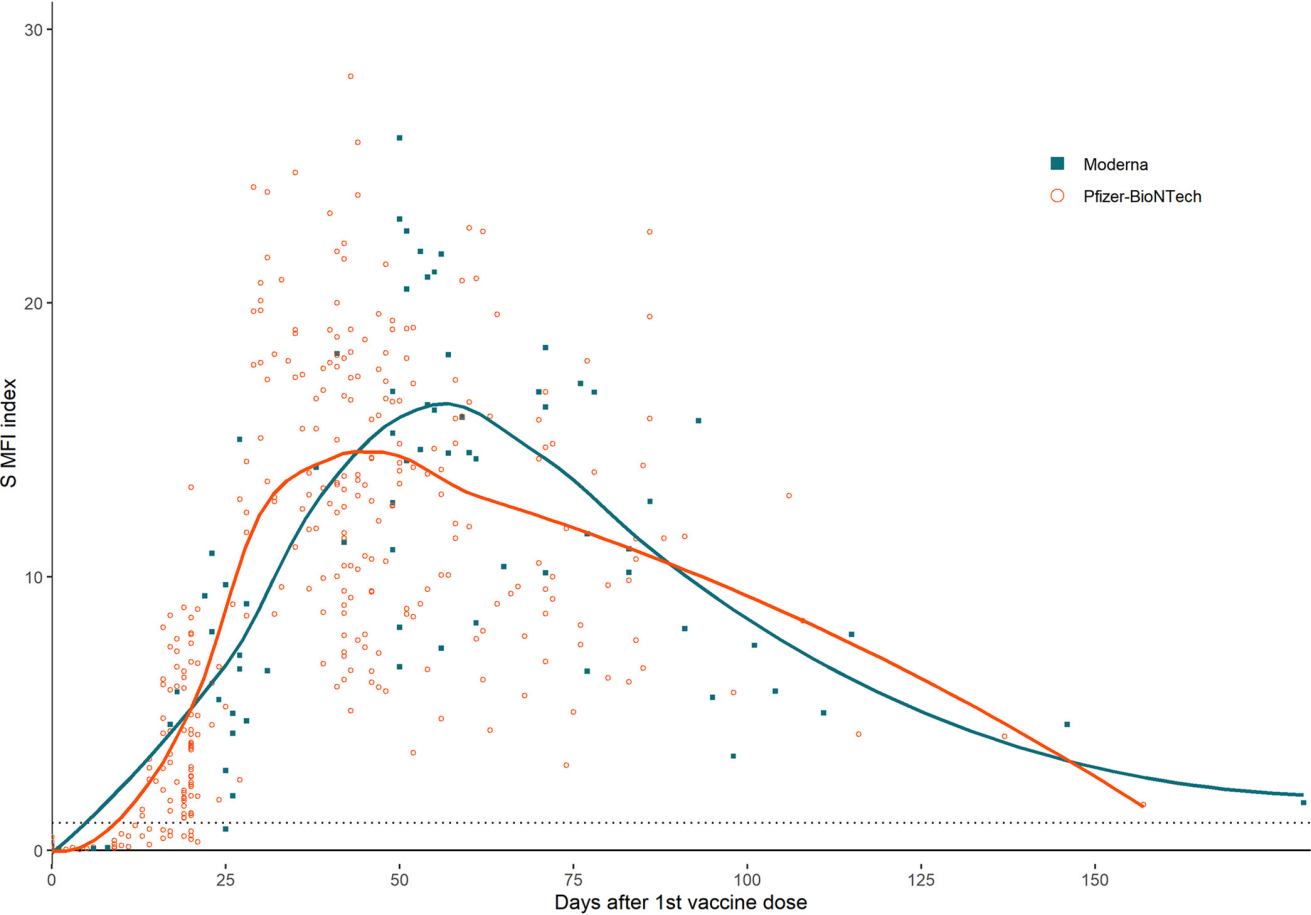
Spike MFI index values versus days post first vaccine dose by vaccine manufacturer. Figure includes only naive patients. Dotted line indicates reactive cutoff. Solid lines are LOESS regressions.

Pfizer-BioNTech vaccine doses are administered 21 days apart while Moderna doses are administered 28 days apart (12, 13). Individuals who received either of these mRNA vaccines showed a steady increase in S antibody levels between their first and second vaccine dose (Pfizer-BioNTech linear regression slope 0 to 21 days post 1st dose = 0.1995, R^2^ = 0.4832, *n* = 178; Moderna linear regression slope 0 to 28 days post 1st dose = 0.2657, R^2^ = 0.5990, *n* = 31) (Fig. 2, Fig. S2). S antibody levels increased rapidly for both groups after receiving their second dose until they reached peak antibody levels (Pfizer-BioNTech linear regression slope 21 to 35 days post 1st dose = 1.130, R^2^= 0.5615, *n* = 44; Moderna linear regression slope 28 to 56 days post 1st dose = 0.3849, R^2^ = 0.2807, *n* = 25). In order to calculate when individuals reached their peak antibody levels after vaccination, S index values from naive individuals were grouped into 1-week bins by days post 1st vaccine dose and the weeks with the highest average index values for each vaccine manufacturer were considered the week of peak antibody levels. Individuals receiving the Moderna vaccine reached peak antibody levels 4 weeks (50 to 56 days post 1st dose) after receiving the second dose of the vaccine (mean S index = 17.43, *n* = 15) while individuals receiving Pfizer-BioNTech reached peak levels 2 weeks (29 to 35 days post 1st dose) after the second dose (mean S index = 17.62, *n* = 25). Peak antibody levels of each group were similar (U = 200, *P* = 0.74). Antibody levels in both groups slowly declined after this peak.

### Effect of age and sex on S antibody levels.

**FIG 3:**
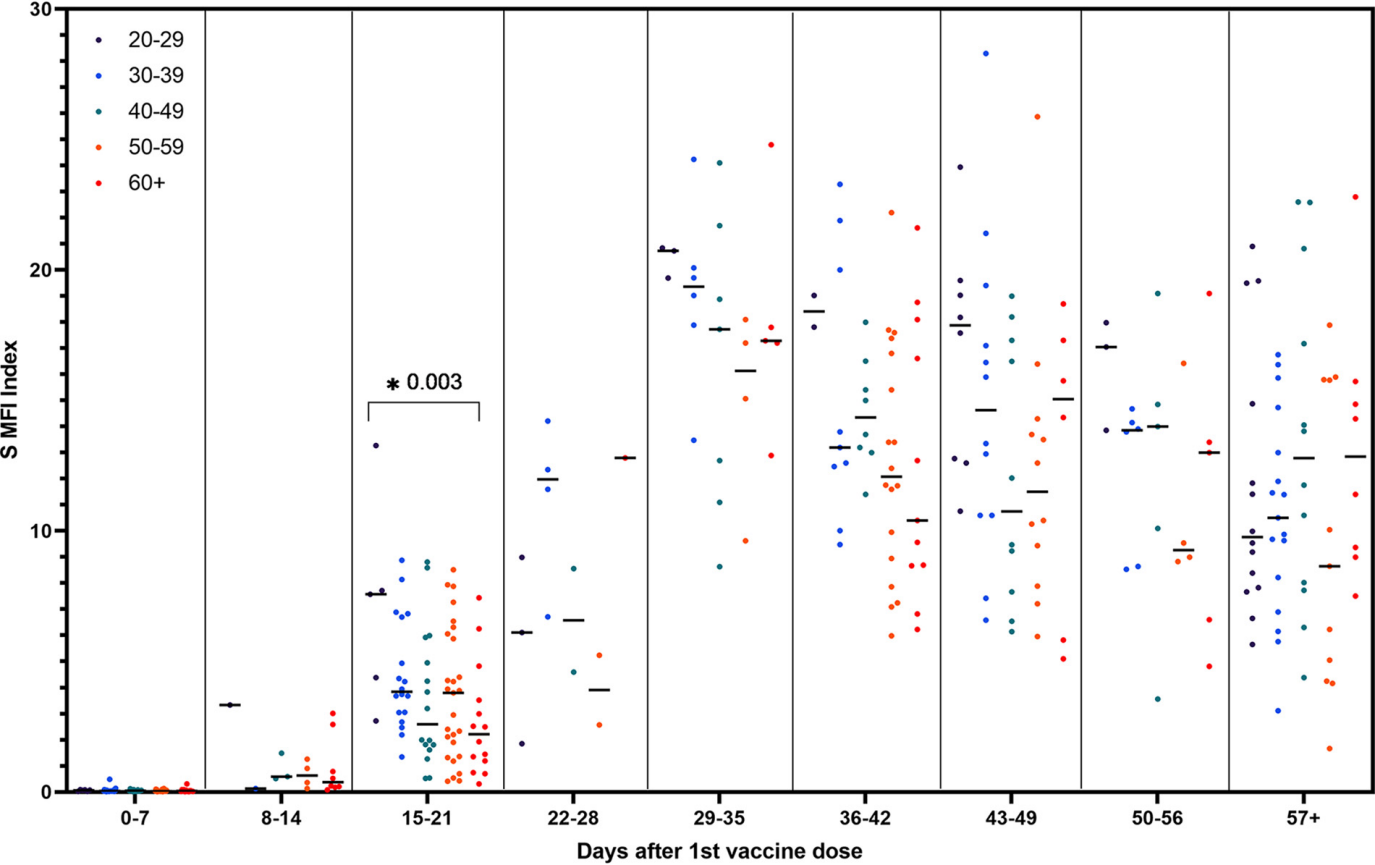
Spike MFI index values in 7-day groups post first vaccination for naive individuals who received the Pfizer-BioNTech vaccine grouped by age group (*n* = 133). *, *P* < 0.05.

**FIG 4:**
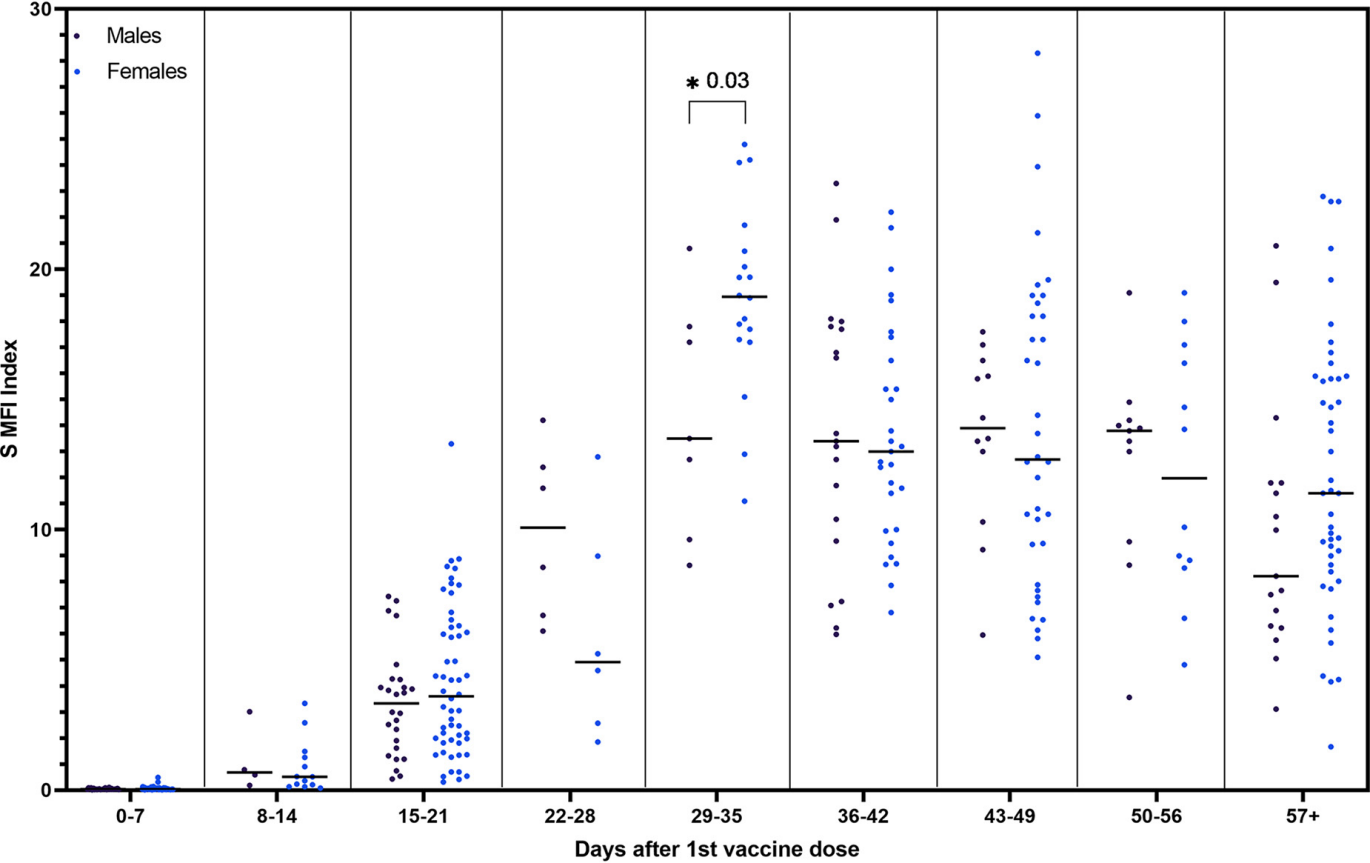
Spike index values in 7-day groups post first vaccination for naive individuals who received the Pfizer-BioNTech vaccine grouped by sex (*n* = 133). *, *P* < 0.05.

In order to assess the effect of age and sex on S antibody response to mRNA vaccination, samples were grouped into 7-day periods based on days after the first vaccine dose. Due to the difference in timing between vaccines and the low number of individuals who received the Moderna vaccine in our study, only data from naive individuals who received the Pfizer-BioNTech vaccine were included in this analysis. No consistent trend among age groups was found across time periods; however, 15 to 21 days after the first vaccine dose, individuals 20 to 29 years old had significantly higher S antibody index values than individuals aged 60 years and older (U = 76, *P* = 0.003) (Fig. 3). Similarly, no consistent difference between males and females was found across time periods. Females had significantly higher S antibody levels 29 to 35 days after their first vaccine dose, or 8 to 14 days after their second dose, compared to males (U = 99, *P* = 0.03) (Fig. 4).

### mRNA vaccine side effects.

We also analyzed the side effects reported by study participants. Local side effects include all side effects near the injection site, including injection site pain, sore arm, redness, and swelling. Systemic side effects include side effects that impacted the whole body or areas of the body not near the injection site. Of all individuals receiving either mRNA vaccine, local side effects were experienced more frequently after the first dose (54%) than after the second (40%, *P* = 0.02) and systemic side effects were experienced more frequently after the second dose (69%) than after the first (31%, *P* < 0.0001) (Table 3). For dose 1, significantly more local side effects were reported than systemic side effects (*P* < 0.0001) while for dose 2, significantly more systemic side effects were reported than local side effects (*P* < 0.0001). Fatigue, muscle or joint pain, and headache were the most commonly reported side effects to either dose.

**TABLE 3:** Frequency of reported side effects for all participants who received an mRNA vaccine

Side effect	Dose 1 count (%)^*a*^	Dose 2 count (%)	*P* value
Any side effect	116 (70)	132 (80)	0.06
Any systemic side effect	52 (31)	114 (69)	<0.0001
Local side effect	89 (54)	67 (40)	0.02
Fatigue	24 (14)	72 (43)	<0.0001
Muscle or joint pain	12 (7)	55 (33)	<0.0001
Headache	18 (11)	48 (29)	<0.0001
Fever	2 (1)	35 (21)	<0.0001
Chills	3 (2)	23 (14)	<0.0001
Nausea or vomiting	2 (1)	13 (8)	0.006
Flu like symptoms	0 (0)	6 (4)	0.03
Lymph node pain or swelling	2 (1)	5 (3)	0.45
Lightheaded or dizzy	3 (2)	4 (2)	>0.9999
Brain Fog	0 (0)	4 (2)	0.12
Congestion	0 (0)	2 (1)	0.50

a Comparisons between the first and second dose were made using Fisher’s exact test.

**FIG 5:**
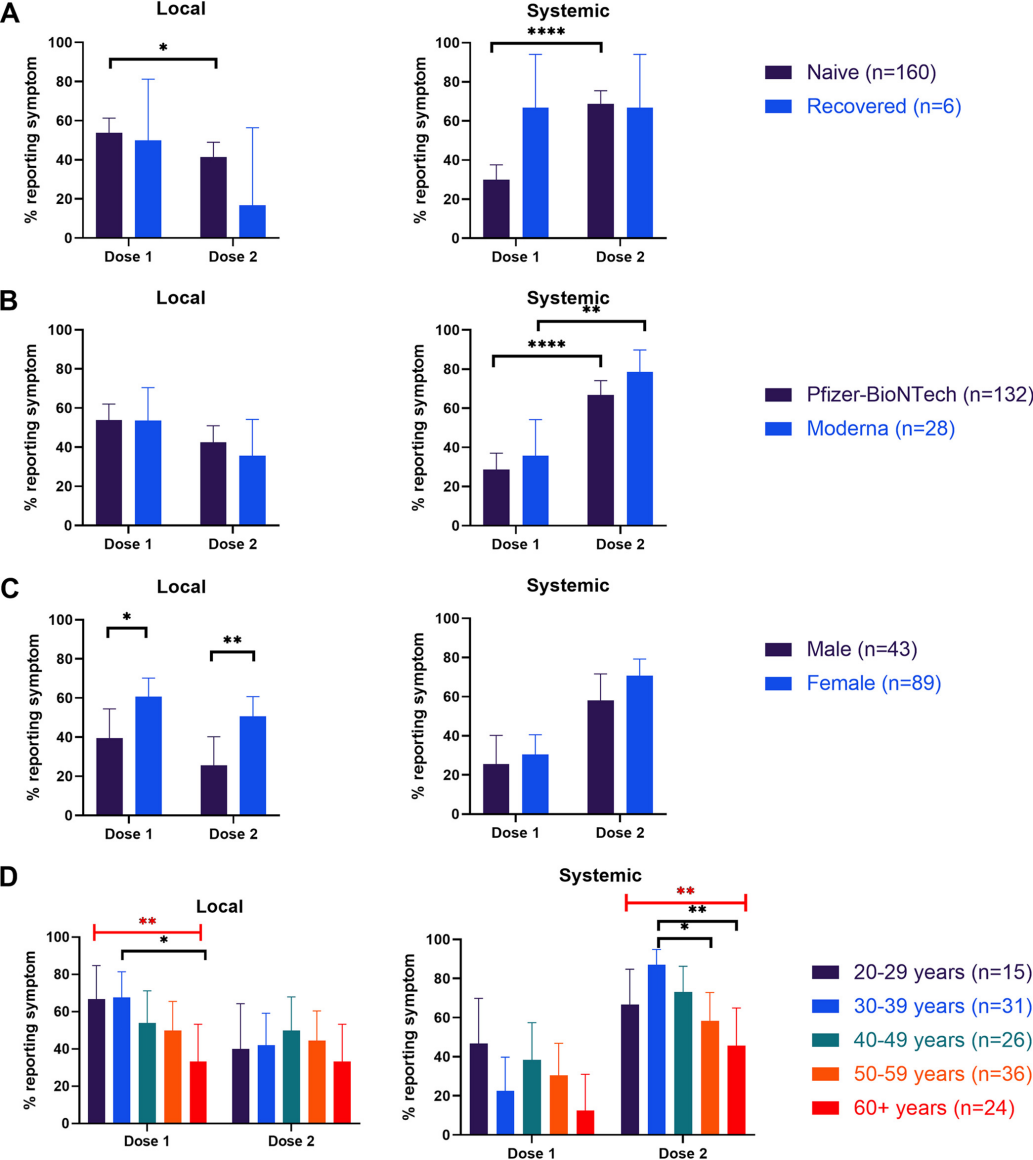
Frequency of local or systemic side effect reporting per vaccine dose for (A) all naive versus recovered individuals who received either mRNA vaccine (B) naive individuals who received Pfizer-BioNTech versus Moderna, (C) naive males versus females who received the Pfizer-BioNTech vaccine, and (D) naive individuals of different age groups who received the Pfizer-BioNTech vaccine. Black brackets indicate pairwise comparisons made using Fisher’s exact test and red brackets indicate comparisons across multiple groups made using Chi-squared test for trend. *, *P* < 0.05; **, *P* < 0.01; ****, *P* < 0.0001.

Although differences in local and systemic side effects were not significant between the naive and recovered groups, recovered individuals reported systemic side effects from dose 1 at a higher frequency than naive individuals and at a similar frequency to what both groups reported for dose 2 (Fig. 5A). There was no significant difference in side effects reported from either dose between naive individuals receiving the Moderna vaccine and naive individuals receiving Pfizer-BioNTech, although individuals receiving the Moderna vaccine reported slightly higher systemic side effects to both doses than those receiving Pfizer-BioNTech (Fig. 5B). Due to the low number of recovered individuals and individuals who received the Moderna vaccine in our study, the influence of age and sex on vaccine side effects were only assessed for naive individuals who received the Pfizer-BioNTech vaccine. Slight differences in local side effect reporting were observed between males and females (dose 1 *P* < 0.05, dose 2 *P* < 0.01) (Fig. 5C). Females reported slightly more systemic side effects than males for both doses, although this difference is not significant. A significant trend was found across age groups for local side effects from dose 1 (*P* < 0.01) where the 20- to 29-year-old and 30- to 39-year-old groups tended to report more side effects than other age groups (Fig. 5D). A similar trend was found for systemic effects experienced after dose 2 (*P* < 0.01) where individuals 30 to 39 years old reported side effects at the highest frequency followed by individuals 40 to 49 years old and 20 to 29 years old.

Finally, we wanted to assess if having a high level of S antibodies after the first vaccine dose was associated with a change in side effects experienced from the second vaccine dose. To do this, we utilized data from all naive individuals who received the Pfizer-BioNTech vaccine and submitted a sample within 1 week prior to receiving their second dose (*n* = 80). We then used Mann-Whitney U Tests to compare the mean MFI index values between individuals who reported side effects and those who did not. Individuals who reported experiencing any side effects had a higher mean S index than individuals not reporting side effects (4.32 versus 2.62, respectively, U = 351, *P* = 0.006). Individuals who reported experiencing muscle or joint pain, fever, nausea or vomiting, and lymph node pain or swelling had significantly higher mean S index values than individuals who did not report those side effects (Table 4).

**TABLE 4:** Mean S MFI index values of individuals who received the Pfizer-BioNTech vaccine and submitted a sample within 1 week before second vaccine dose based on side effects reported from the second dose of the vaccine (*n* = 80)^*a*^

Side effect	# reporting side effect	Mean index values of individuals not reporting side effect	Mean index values of individuals reporting side effect	*P*-value
Any side effect	60	2.62	4.32	0.006
Any systemic side effect reported	56	3.39	4.12	0.30
Local side effect	33	3.74	4.12	0.33
Fatigue	35	3.65	4.21	0.63
Muscle or joint pain	26	3.21	5.32	0.0003
Headache	20	3.66	4.61	0.29
Fever	17	3.50	5.36	0.023
Chills	13	3.80	4.43	0.54
Nausea or vomiting	7	3.66	6.43	0.030
Lymph node pain or swelling	3	3.75	7.72	0.017
Congestion	3	3.88	4.27	0.90
Flu-like symptoms	3	3.85	5.16	0.39
Lightheaded or dizzy	2	3.89	4.32	0.48
Brain fog	2	3.86	5.50	0.43

a Comparisons were made using Mann-Whitney U tests.

## DISCUSSION

As COVID-19 vaccines became authorized in the United States, establishing a mechanism to capture blood samples before and after vaccination for serological assessment became an urgent need for our public health laboratory. Our ability to initiate a study within the relatively brief window between vaccine authorization and distribution was facilitated by recruiting laboratory staff to self-collect DBS specimens before and after receiving vaccine doses. We enrolled 175 individuals who submitted 509 DBS, 507 (99.6%) of which were suitable for testing. While 14% of individuals reported issues with DBS collection, none of these issues prevented the DBS from being tested. This success supports the potential for self-collected DBS to be used as an effective serosurvey strategy to assess response to vaccination or general antibody prevalence. However, our study participants were primarily laboratory personnel and included many individuals who are familiar with DBS. Therefore, this high rate of success in DBS collection may not occur in the general population. Similar studies using at-home self-collected DBS reported lower return rates than this study (10, 14–16) but the proportion of samples that were unsuitable for testing upon arriving at the lab were comparable (14–16). It is of note that while our study demonstrates an effective use of self-collected DBS as a sample type, rather than more invasive and labor-intensive plasma and serum samples, the test still requires trained laboratory staff, specialized equipment, and time to perform. However, the test used in this study can be performed in less than a day and is designed to accommodate high-throughput testing, resulting in a short turnaround time in the lab. Additionally, not including labor and equipment costs, this assay has a relatively low reagent cost of approximately $1.00 per sample (4, 5).

Using DBS, we saw that vaccinated individuals’ spike IgG antibody levels increased until several weeks after their last dose before beginning to slowly decline, matching results from other studies that used serum/plasma (17–19). Vaccinated individuals’ spike IgG also reached higher levels than were seen in those who were infected but not yet vaccinated (18, 20–26). Also, like previous studies, we found that the seven recovered individuals in our study showed an abrupt increase in antibodies after the first dose of vaccine while naive individuals showed a more gradual increase in antibody levels (18, 21–23, 25–29). However, due to the small number of recovered individuals in our study, we cannot generalize our results regarding the difference in final antibody levels or rate of antibody waning between naive and previously infected individuals.

Naïve individuals who received the Moderna or Pfizer-BioNTech vaccine reached their peak antibody levels at different times which may be partially associated with the difference in dosage timing. Our study found that both groups receiving an mRNA vaccine reached similar peak antibody levels following their second dose. Data from some studies support this finding (20, 30); however, other studies have found that individuals who received the Moderna vaccine reached higher antibody levels than those who received the Pfizer-BioNTech vaccine (19, 31, 32). These different findings may be due to sample size effects and differences between sampled populations.

Our study did not find any consistent trend of S antibody levels induced by the Pfizer-BioNTech vaccine by age group. However, our study and several others found that younger age groups reached higher antibody levels between the first and second doses (28, 30, 31, 33, 34). Other studies have also found that younger individuals reach higher antibody levels than older individuals following their second dose (18, 24, 32, 35). Although Amodio et al. found that females reached higher IgG concentrations than males (24), in agreement with other work, we did not find a consistent correlation between sex and S antibody levels (30, 33–35). Due to a relatively small male sample size in our study, it is difficult to draw conclusions regarding sex differences in S IgG concentrations.

Our investigation of the number of side effects reported by individuals who received either mRNA vaccine did not reveal a significant difference between individuals who received the Moderna or Pfizer-BioNTech vaccine. However, other studies have shown that individuals who received the Moderna vaccine reported more side effects than those who received the Pfizer-BioNTech vaccine, suggesting that our data may have approached statistical significance if our study population included more individuals who received the Moderna vaccine (19, 36, 37). We found that recovered individuals reported more systemic side effects after receiving their first dose of the vaccine than naive individuals did and although these results did not reach statistical significance, several other studies have found a similar pattern (23, 25, 26, 38, 39).

Our analysis of naive individuals who received either mRNA vaccine showed that the most commonly reported side effects in our study, injection site pain, fatigue, muscle or joint pain, and headache, are similar to those reported in other studies and clinical trial data (12, 13, 18, 20, 23, 37, 40). Our study found similar rates of both local and systemic side effects to several studies (23, 26, 38); however, clinical trials and other studies have reported higher frequencies of both systemic and local side effects (12, 13, 36, 37, 39). Similar to some studies, we found that the occurrence of local side effects decreased from the first to the second dose while the occurrence of systemic side effects increased (13, 39). However, other studies have found that the rate of local side effects increases from dose 1 to dose 2 (12, 18, 25). Our questionnaire used a free response write-in section for participants to describe their side effects. This free response strategy may have resulted in individuals forgetting to describe certain symptoms because they were not prompted or did not deem them significant enough to list resulting in skewed data.

Within the Pfizer vaccinated group, we saw significant differences in side effects experienced by age group. Younger age groups were more likely to report local side effects after dose 1 than older groups. After dose 2, individuals 30 to 39 years old reported systemic side effects at the highest rates, followed by individuals 40 to 49 years old and 20 to 29 years old. Clinical trials and similar studies have found that younger individuals tend to experience more side effects from COVID-19 vaccination than older individuals (13, 39–41). We also found that females were more likely to experience side effects than males where the difference in local side effects was statistically significant and the difference in systemic side effects was not. Other authors have also found that females are more likely to experience side effects than males (39, 40); although, Klugar et al. reported that both sexes experienced similar levels of side effects (41). We found that individuals with higher antibody levels in the week prior to dose 2 reported more side effects after dose 2 than those with lower antibody titers. Michos et al. found a similar result when comparing antibody levels after the second dose while Röltgen et al. did not find a correlation between antibody levels and side effects experienced (18, 33).

The major limitations of our study are related to the use of a convenience sample which does not reflect the general population. Study participants were highly motivated due to their involvement in public health work, highly educated, actively employed, presumed healthy, and lacked diversity. We also do not have data from recruited individuals who decided not to participate. The study population was mostly female, between the ages of 40 and 59, White, not Hispanic or Latino, and had not been previously infected with SARS-CoV-2. Additionally, the majority of participants received the Pfizer-BioNTech vaccine and data on those who received the Moderna or Janssen vaccine is limited. We also relied on self-reports and a nonstandard, write-in method to collect information on side effects from the vaccine which required an individual to manually categorize side effects and leaves room for misinterpretation of responses. Individuals were enrolled in the study at various times before and after vaccination, so this study does not have a standard sample follow-up period. Finally, this study did not include measurements of neutralizing antibodies or other indicators of humoral immune response. While antibody levels have been correlated with neutralizing antibody levels (33, 42, 43), antibody levels alone should not be used to make predictions about the ability of different vaccines to protect against SARS-CoV-2 infection or disease.

### Conclusion.

We demonstrate that self-collected DBS and a multiplex MIA are an effective way to measure antibody responses to COVID-19 vaccines. Our results using DBS were similar to previous vaccine response studies that used serum/plasma samples. Self-collected DBS are a convenient option for obtaining blood specimens to monitor antibody response to vaccinations and could also be used to perform large-scale population serosurveys for SARS-CoV-2 or other emerging infections.

## MATERIALS AND METHODS

### Recruitment and sample collection.

Starting in February 2021, we enrolled a convenience sample of current employees, students, or fellows at the Wadsworth Center, NYS Department of Health (DOH) who were at least 18 years old and planned on receiving, or had recently received, a COVID-19 vaccine. Participants were recruited using fliers emailed to their DOH email address. Approval for human subject research was obtained from the New York State Department of Health Institutional Review Board (IRB) (21-002).

Eligible individuals who signed and returned an informed consent form received a DBS collection kit which contained instructions for collecting DBS, three preaddressed envelopes, three postage stamps, six safety lancets, six gauze pads, six alcohol wipes, and six bandages. The kits also contained three questionnaires for collecting demographic information, COVID-19 infection history, previous COVID-19 antibody test history, vaccination details and side effects, and three 903 five-spot blood collection cards (Eastern Business Forums, Maudlin, SC) each labeled with a barcoded and deidentified participant ID. Participants completed and returned a questionnaire with each DBS card. The instructions included photographs and text describing precollection set-up, collection, and postcollection procedures (Fig. S1).

Participants collected a total of three DBS and followed one of two collection schedules based on their vaccination status at enrollment. Individuals who were not vaccinated prior to enrollment collected samples 1 to 7 days before the first dose of vaccine, 1 to 7 days before the second dose of vaccine, or 3 to 4 weeks after the first DBS collection, and 3 to 4 weeks after the second DBS collection. Individuals who were vaccinated prior to enrollment collected a sample upon receiving the kit, 3 to 4 weeks after the first DBS collection, and 3 to 4 weeks after the second DBS collection. Participants were sent emails 2 to 3 weeks after the first and second DBS were received to remind them to collect their next DBS. If follow-up samples were not submitted, participants received one additional reminder email. Participants placed the DBS card and the accompanying questionnaire in the preaddressed envelopes and either mailed them to the laboratory or dropped them off in assigned locations. Upon receipt, laboratory staff labeled each DBS card with a unique accessioning barcode and checked the questionnaires and cards for any issues. Samples received more than 14 days after collection were marked as unsuitable for testing (*n* = 2). Participants were notified if their sample was unsuitable and allowed to collect and submit another sample.

Following completion or withdrawal from the study, participants were sent a report of their results which included the individual's nucleocapsid and spike index values, qualitative result, and interpretation for each DBS collected. The report also included the criteria used to determine qualitative results, information to help interpret the data, and a graph showing the mean spike median fluorescent intensity (MFI) index values by days after first dose of vaccine for all study samples submitted up to that point.

### Assay procedure.

Magplex-C microspheres were coupled as previously described (4). Three bead sets were coupled: a spike S1 subunit antigen from SinoBiological (S) (Beijing, China), and two nucleocapsid antigens, one from SinoBiological (N-SB) and one from Native Antigen (N-NA) (Oxford, UK). DBS cards were punched using Panthera or Wallac punchers (PerkinElmer, Waltham, MA). One 3.2-mm punch was added to each well of a round-bottom, nontreated, polystyrene 96-well plate (Corning, Corning, NY). Punches were then eluted in 250 μL elution buffer (Tris-buffered saline, 1% casein blocker) (Bio-Rad Laboratories, Hercules, CA) for 1 h at room temperature (19°C to 22°C). Beads and eluate (25 μL of each, 1,250 beads/bead set/well) were then transferred to nonbinding 384-well plates (Greiner Bio-One, Monroe, NC) and incubated for 30 min at 37°C and shaken at 300 RPM in the dark. Samples were washed using wash buffer (PBS, 2% BSA, 0.02% Tween, 0.05% azide, pH 7.5) on a BioTek 405 TSUS magnetic microplate washer. After washing, samples were incubated with 50 μL phycoerythrin-tagged goat-anti human IgG at a concentration of 0.4 μg/mL (Thermo Fisher Scientific - Invitrogen-eBiosciences, Waltham, MA or SouthernBiotech, Birmingham, AL). Plates were then incubated for 30 min and washed as previously described. Ninety μL of xMap sheath fluid (Luminex Corp., Austin, TX) were added to each well and plates were shaken at room temperature in the dark at 300 RPM for 1 min to resuspend beads. Assays were performed manually or by an automated Microlab Star liquid handling system (Hamilton Company, Reno, NV) to perform DBS elution, microsphere addition, sample addition, and secondary antibody addition. Plates run using the liquid handlers were incubated on Hamilton Heater Shakers. Samples were analyzed using a FlexMap 3D instrument (Luminex Corp., Austin, TX) and reported as MFI for each bead set.

Cutoff values were established for each bead set based on analysis of 789 known negative newborn and adult DBS samples. The mean MFI +6 standard deviations is considered reactive. Index values were calculated using the sample MFI divided by the reactive cutoff value for each bead set; values of >1.0 indicate a reactive result for that bead set. This assay was validated and approved for clinical testing by New York State Department of Health Clinical Laboratory Evaluation Program.

### Data analysis.

Data were cleaned using Microsoft Excel. Participant’s infection status was determined by self-reported positive PCR test, COVID-19 diagnosis, or a nucleocapsid index value >1. Individuals infected prior to starting the study were assigned to the recovered group and individuals not infected at the start of the study were assigned to the naive group. Self-reported DBS collection issues and side effects were categorized using key words and were manually reviewed by a single analyst.

Comparisons of continuous variables were made using Mann-Whitney U tests for comparing two groups and Kruskal-Wallis tests for multiple comparisons. Comparisons of categorical variables were made using Fishers Exact test for comparing two groups or a chi-squared test for trend for comparing multiple groups. Two tailed *P*-values are reported and *P*-values of <0.05 are considered significant. Figures were created using R (v4.0.5) or GraphPad Prism (v9.2.0).

## References

[1] Metcalf CJE, Farrar J, Cutts FT, Basta NE, Graham AL, Lessler J, Ferguson NM, Burke DS, Grenfell BT. 2016. Use of serological surveys to generate key insights into the changing global landscape of infectious disease. Lancet 388:728–730. doi:10.1016/S0140-6736(16)30164-7. 27059886 PMC5678936

[2] Cutts FT, Hanson M. 2016. Seroepidemiology: an underused tool for designing and monitoring vaccination programmes in low-and middle-income countries Trop Med Int Health 21:1086–1098. doi:10.1111/tmi.12737. 27300255

[3] Rosenberg ES, Dorabawila V, Easton D, Bauer UE, Kumar J, Hoen R, Hoefer D, Wu M, Lutterloh E, Conroy MB, Greene D, Zucker HA. 2022. Covid-19 vaccine effectiveness in New York State. N Engl J Med 386:116–127. doi:10.1056/NEJMoa2116063. 34942067 PMC8693697

[4] Styer LM, Hoen R, Rock J, Yauney E, Nemeth K, Bievenue R, Parker MM. 2021. High-throughput multiplex SARS-CoV-2 IgG microsphere immunoassay for dried blood spots: a public health strategy for enhanced serosurvey capacity. Microbiol Spectr 9. doi:10.1128/Spectrum.00134-21. PMC8552730 34319133

[5] Damjanovic A, Styer LM, Nemeth K, Yauney E, Rock JM, Bievenue R, Hoen R, Ehrbar D, Kay DM, Caggana M, Parker MM. 2022. Utility of newborn dried blood spots to ascertain seroprevalence of SARS-CoV-2 antibodies among individuals giving birth in New York State, November 2019 to November 2021. JAMA Netw Open 5:e2227995. doi:10.1001/jamanetworkopen.2022.27995. 35994287 PMC9396364

[6] Food and Drug Administration. 2020. FDA takes key action in fight against COVID-19 by issuing emergency use authorization for first COVID-19 vaccine | FDA. https://www.fda.gov/news-events/press-announcements/fda-takes-key-action-fight-against-covid-19-issuing-emergency-use-authorization-first-covid-19. Retrieved 14 February 2022.

[7] Food and Drug Administration. 2020. FDA takes additional action in fight against COVID-19 by issuing emergency use authorization for second COVID-19 vaccine | FDA. https://www.fda.gov/news-events/press-announcements/fda-takes-additional-action-fight-against-covid-19-issuing-emergency-use-authorization-second-covid. Retrieved 14 February 2022.

[8] Food and Drug Administration. 2021. FDA issues emergency use authorization for third COVID-19 Vaccine | FDA. https://www.fda.gov/news-events/press-announcements/fda-issues-emergency-use-authorization-third-covid-19-vaccine. Retrieved 14 February 2022.

[9] Valentine-Graves M, Hall E, Guest JL, Adam E, Valencia R, Shinn K, Hardee I, Sanchez T, Siegler AJ, Sullivan PS. 2020. At-home self-collection of saliva, oropharyngeal swabs and dried blood spots for SARS-CoV-2 diagnosis and serology: postcollection acceptability of specimen collection process and patient confidence in specimens. PLoS One 15:e0236775. doi:10.1371/journal.pone.0236775. 32756585 PMC7406082

[10] Tang X, Sharma A, Pasic M, Colwill K, Birnboim C, Nagelkerke N, Bogoch I, Schultz C, Newcombe L, Slater J, Rodriguez P, Huang G, Fu SH, Meh C, Wu CN, Kaul R, Langlois M-A, Morawski E, Hollander A, Eliopoulos D, Aloi B, Lambe T, Abe K, Caldwell L, Barrios-Rodiles M, Fazel-Zarandi M, Weingust R, Wang J, Rathod B, Santhanam DR, Cho ER, Qu K, Jha S, Jha V, Suraweera W, Wen R, Sinha S, Reid A, Gingras A-C, Chakraborty P, Slutsky AS, Jha P. 2021. COVID symptoms, seroprevalence, and mortality during the first wave of SARS-CoV-2 in Canada. SSRN Electronic J. 1–38.

[11] McDade TW, Demonbreun AR, Sancilio A, Mustanski B, D’Aquila RT, McNally EM. 2021. Durability of antibody response to vaccination and surrogate neutralization of emerging variants based on SARS-CoV-2 exposure history. Sci Rep 11:1–6. doi:10.1038/s41598-021-96879-3. 34462501 PMC8405730

[12] Baden LR, El Sahly HM, Essink B, Kotloff K, Frey S, Novak R, Diemert D, Spector SA, Rouphael N, Creech CB, McGettigan J, Khetan S, Segall N, Solis J, Brosz A, Fierro C, Schwartz H, Neuzil K, Corey L, Gilbert P, Janes H, Follmann D, Marovich M, Mascola J, Polakowski L, Ledgerwood J, Graham BS, Bennett H, Pajon R, Knightly C, Leav B, Deng W, Zhou H, Han S, Ivarsson M, Miller J, Zaks T. 2021. Efficacy and safety of the mRNA-1273 SARS-CoV-2 vaccine. N Engl J Med 384:403–416. doi:10.1056/NEJMoa2035389. 33378609 PMC7787219

[13] Polack FP, C4591001 Clinical Trial Group, Thomas SJ, Kitchin N, Absalon J, Gurtman A, Lockhart S, Perez JL, Pérez Marc G, Moreira ED, Zerbini C, Bailey R, Swanson KA, Roychoudhury S, Koury K, Li P, Kalina WV, Cooper D, Frenck RW, Hammitt LL, Türeci Ö, Nell H, Schaefer A, Ünal S, Tresnan DB, Mather S, Dormitzer PR, Şahin U, Jansen KU, Gruber WC. 2020. Safety and efficacy of the BNT162b2 mRNA Covid-19 vaccine. N Engl J Med 383:2603–2615. doi:10.1056/NEJMoa2034577. 33301246 PMC7745181

[14] Hirshfield S, Teran RA, Downing MJ, Chiasson MA, Van Tieu H, Dize L, Gaydos CA. 2018. Quantification of HIV-1 RNA among men who have sex with men using an at-home self-collected dried blood spot specimen: feasibility study. JMIR Public Health Surveill 4. doi:10.2196/10847. PMC6238105 30389648

[15] Roberts SC, Seav SM, McDade TW, Dominick SA, Gorman JR, Whitcomb BW, Su HI. 2016. Self-collected dried blood spots as a tool for measuring ovarian reserve in young female cancer survivors. Hum Reprod 31:1570–1578. doi:10.1093/humrep/dew114. 27170433 PMC4901885

[16] Luo W, Sullivan V, Chavez PR, Wiatrek SE, Zlotorzynska M, Martin A, Rossetti R, Sanchez T, Sullivan P, MacGowan RJ, Owen SM, Masciotra S. 2021. The feasibility of modified HIV and antiretroviral drug testing using self-collected dried blood spots from men who have sex with men. BMC Infect Dis 21:1–7. doi:10.1186/s12879-021-06110-x. 33952212 PMC8098001

[17] Wisnewski AV, Luna JC, Redlich CA. 2021. Human IgG and IgA responses to COVID-19 mRNA vaccines. PLoS One 16:e0249499. doi:10.1371/journal.pone.0249499. 34133415 PMC8208542

[18] Röltgen K, Nielsen SCA, Arunachalam PS, Yang F, Hoh RA, Wirz OF, Lee AS, Gao F, Mallajosyula V, Li C, Haraguchi E, Shoura MJ, Wilbur JL, Wohlstadter JN, Davis MM, Pinsky BA, Sigal GB, Pulendran B, Nadeau KC, Boyd SD. 2021. mRNA vaccination compared to infection elicits an IgG-predominant response with greater SARS-CoV-2 specificity and similar decrease in variant spike recognition. medRxiv.

[19] Bliden KP, Liu T, Sreedhar D, Kost J, Hsiung J, Zhao S, Shan D, Usman A, Walia N, Cho A, Jerjian C, Tantry US, Gurbel PA, Tang M, Dai H. 2021. Evolution of anti-SARS-CoV-2 IgG antibody and IgG avidity post Pfizer and Moderna mRNA vaccinations. medRxiv.

[20] Iddins BO, Buck B, Cato T, Partin A, Attia K, Wesh C, Shourbaji R, Waugh MH. 2021. mRNA SARS-CoV-2 immunization confers robust antibody response in occupational healthcare workers and fosters workplace safety. J Occup Environ Med 63:E314–E317. doi:10.1097/JOM.0000000000002187. 33928943 PMC8091905

[21] Azzi L, Focosi D, Dentali F, Baj A, Maggi F. 2021. Anti-SARS-CoV-2 RBD IgG responses in convalescent versus naïve BNT162b2 vaccine recipients. Vaccine 39:2489–2490. doi:10.1016/j.vaccine.2021.03.086. 33824042 PMC8009041

[22] Ciccone EJ, Zhu DR, Ajeen R, Lodge EK, Shook-Sa BE, Boyce RM, Aiello AE. 2021. SARS-CoV-2 seropositivity after infection and antibody response to mRNA-based vaccination. medRxiv.

[23] Krammer F, Srivastava K, Alshammary H, Amoako AA, Awawda MH, Beach KF, Bermúdez-González MC, Bielak DA, Carreño JM, Chernet RL, Eaker LQ, Ferreri ED, Floda DL, Gleason CR, Hamburger JZ, Jiang K, Kleiner G, Jurczyszak D, Matthews JC, Mendez WA, Nabeel I, Mulder LCF, Raskin AJ, Russo KT, Salimbangon A-BT, Saksena M, Shin AS, Singh G, Sominsky LA, Stadlbauer D, Wajnberg A, Simon V. 2021. Antibody responses in seropositive persons after a single dose of SARS-CoV-2 mRNA vaccine. N Engl J Med 384:1372–1374. doi:10.1056/NEJMc2101667. 33691060 PMC8008743

[24] Amodio E, Capra G, Casuccio A, De Grazia S, Genovese D, Pizzo S, Calamusa G, Ferraro D, Giammanco GM, Vitale F, Bonura F. 2021. Antibodies responses to SARS-CoV-2 in a large cohort of vaccinated subjects and seropositive patients. Vaccines (Basel) 9. doi:10.3390/vaccines9070714. PMC8309986 34358130

[25] Ebinger JE, Fert-Bober J, Printsev I, Wu M, Sun N, Figueiredo JC, Van Eyk JE, Braun JG, Cheng S, Sobhani K. 2021. Prior COVID-19 infection and antibody response to single versus double dose mRNA SARS-CoV-2 vaccination. medRxiv.

[26] Ontañón J, Blas J, de Cabo C, Santos C, Ruiz-Escribano E, García A, Marín L, Sáez L, Beato JL, Rada R, Navarro L, Sainz de Baranda C, Solera J, Sáez L, Solera J. 2021. Influence of past infection with SARS-CoV-2 on the response to the BNT162b2 mRNA vaccine in health care workers: kinetics and durability of the humoral immune response. EBioMedicine 73:103656–103656. doi:10.1016/j.ebiom.2021.103656. 34740112 PMC8556513

[27] Gobbi F, Buonfrate D, Moro L, Rodari P, Piubelli C, Caldrer S, Riccetti S, Sinigaglia A, Barzon L. 2021. Antibody response to the BNT162b2 mRNA COVID-19 vaccine in subjects with prior SARS-CoV-2 infection. Viruses 13:422–10. doi:10.3390/v13030422. 33807957 PMC8001674

[28] Prendecki M, Clarke C, Brown J, Cox A, Gleeson S, Guckian M, Randell P, Pria AD, Lightstone L, Xu XN, Barclay W, McAdoo SP, Kelleher P, Willicombe M. 2021. Effect of previous SARS-CoV-2 infection on humoral and T-cell responses to single-dose BNT162b2 vaccine. Lancet 397:1178–1181. doi:10.1016/S0140-6736(21)00502-X. 33640037 PMC7993933

[29] Samanovic AMI, Cornelius AR, Gray-Gaillard SL, Allen JR, Karmacharya T, Wilson JP, Wesley S, Tuen M, Koralov SB, Mulligan MJ, Sedaghat R. 2021. Robust immune responses after one dose of BNT162b2 mRNA vaccine dose in SARS-CoV-2 experienced individuals. medRxiv. 10.1126/scitranslmed.abi8961 PMC9248013 34874183

[30] Wheeler SE, Shurin GV, Yost M, Anderson A, Pinto L, Wells A, Shurin MR. 2021. Differential antibody response to mRNA COVID-19 vaccines in healthy subjects. Microbiol Spectr 9. doi:10.1128/Spectrum.00341-21. PMC8552678 34346750

[31] Richards NE, Keshavarz B, Workman LJ, Nelson MR, Platts-Mills TAE, Wilson JM. 2021. Comparison of SARS-CoV-2 antibody response by age among recipients of the BNT162b2 vs the mRNA-1273 vaccine. JAMA Netw Open 4:e2124331. doi:10.1001/jamanetworkopen.2021.24331. 34473262 PMC8414189

[32] Steensels D, Pierlet N, Penders J, Mesotten D, Heylen L. 2021. Comparison of SARS-CoV-2 antibody response following vaccination with BNT162b2 and mRNA-1273. JAMA - JAMA 326:1533–1535. doi:10.1001/jama.2021.15125. 34459863 PMC8406205

[33] Michos A, Tatsi EB, Filippatos F, Dellis C, Koukou D, Efthymiou V, Kastrinelli E, Mantzou A, Syriopoulou V. 2021. Association of total and neutralizing SARS-CoV-2 spike -receptor binding domain antibodies with epidemiological and clinical characteristics after immunization with the 1st and 2nd doses of the BNT162b2 vaccine. Vaccine 39:5963–5967. doi:10.1016/j.vaccine.2021.07.067. 34400017 PMC8302834

[34] Terpos E, Trougakos IP, Apostolakou F, Charitaki I, Sklirou AD, Mavrianou N, Papanagnou ED, Liacos CI, Gumeni S, Rentziou G, Korompoki E, Papassotiriou I, Dimopoulos MA. 2021. Age-dependent and gender-dependent antibody responses against SARS-CoV-2 in health workers and octogenarians after vaccination with the BNT162b2 mRNA vaccine. Am J Hematol 96:E257–E259. doi:10.1002/ajh.26185. 33837984 PMC8250071

[35] Dörschug A, Frickmann H, Schwanbeck J, Yilmaz E, Mese K, Hahn A, Groß U, Zautner AE. 2021. Comparative assessment of sera from individuals after S-gene RNA-based SARS-CoV-2 vaccination with spike-protein-based and nucleocapsid-based serological assays. Diagnostics 11:426. doi:10.3390/diagnostics11030426. 33802453 PMC7998789

[36] Kadali RAK, Janagama R, Peruru S, Gajula V, Madathala RR, Chennaiahgari N, Malayala SV. 2021. Non-life-threatening adverse effects with COVID-19 mRNA-1273 vaccine: a randomized, cross-sectional study on healthcare workers with detailed self-reported symptoms. J Med Virol 93:4420–4429. doi:10.1002/jmv.26996. 33822361 PMC8250701

[37] Kadali RAK, Janagama R, Peruru S, Malayala SV. 2021. Side effects of BNT162b2 mRNA COVID-19 vaccine: a randomized, cross-sectional study with detailed self-reported symptoms from healthcare workers. Int J Infect Dis 106:376–381. doi:10.1016/j.ijid.2021.04.047. 33866000 PMC8049195

[38] Tissot N, Brunel AS, Bozon F, Rosolen B, Chirouze C, Bouiller K. 2021. Patients with history of COVID-19 had more side effects after the first dose of COVID-19 vaccine. Vaccine 39:5087–5090. doi:10.1016/j.vaccine.2021.07.047. 34332800 PMC8295016

[39] Menni C, Klaser K, May A, Polidori L, Capdevila J, Louca P, Sudre CH, Nguyen LH, Drew DA, Merino J, Hu C, Selvachandran S, Antonelli M, Murray B, Canas LS, Molteni E, Graham MS, Modat M, Joshi AD, Mangino M, Hammers A, Goodman AL, Chan AT, Wolf J, Steves CJ, Valdes AM, Ourselin S, Spector TD. 2021. Vaccine side-effects and SARS-CoV-2 infection after vaccination in users of the COVID Symptom Study app in the UK: a prospective observational study. Lancet Infect Dis 21:939–949. doi:10.1016/S1473-3099(21)00224-3. 33930320 PMC8078878

[40] Riad A, Hocková B, Kantorová L, Slávik R, Spurná L, Stebel A, Havriľak M, Klugar M. 2021. Side effects of mRNA-based COVID-19 vaccine: nationwide phase IV study among healthcare workers in Slovakia. Pharmaceuticals 14:873–24. doi:10.3390/ph14090873. 34577573 PMC8466035

[41] Klugar M, Riad A, Mekhemar M, Conrad J, Buchbender M, Howaldt HP, Attia S. 2021. Side effects of MRNA-based and viral vector-based COVID-19 vaccines among German healthcare workers. Biology (Basel) 10:752–21. doi:10.3390/biology10080752. 34439984 PMC8389568

[42] Lee WT, Girardin RC, Dupuis AP, Kulas KE, Payne AF, Wong SJ, Arinsburg S, Nguyen FT, Mendu DR, Firpo-Betancourt A, Jhang J, Wajnberg A, Krammer F, Cordon-Cardo C, Amler S, Montecalvo M, Hutton B, Taylor J, Mcdonough KA. 2021. Neutralizing antibody responses in COVID-19 convalescent sera. J Infect Dis 223:47–55. doi:10.1093/infdis/jiaa673. 33104179 PMC7665673

[43] Muecksch F, Wise H, Batchelor B, Squires M, Semple E, Richardson C, McGuire J, Clearly S, Furrie E, Greig N, Hay G, Templeton K, Lorenzi JCC, Hatziioannou T, Jenks S, Bieniasz PD. 2021. Longitudinal serological analysis and neutralizing antibody levels in coronavirus disease 2019 convalescent patients. J Infect Dis 223:389–398. doi:10.1093/infdis/jiaa659. 33140086 PMC7665595

