# Supplementary material for: Use of Self-Collected Dried Blood Spots and a Multiplex Microsphere Immunoassay to Measure IgG Antibody Response to COVID-19 Vaccines
Source: Microbiol Spectr. 2023 Jan 9;11(1):e01336-22. doi: 10.1128/spectrum.01336-22 (PMC9927373; doi:10.1128/spectrum.01336-22)
Supplement: Supplemental file 1 — Supplemental material. Download spectrum.01336-22-s0001.pdf, PDF file, 0.5 MB [file spectrum.01336-22-s0001.pdf]

Supplemental Fig 1. DBS Collection Instructions

Instructions for Self-Collection of Dried Blood Spots (DBS)

This kit contains supplies to collect 3 DBS : 3 blood collection cards 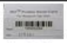 , 3 envelopes, 3 questionnaires, 6 safety lancets 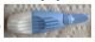 , 6 gauze pads 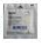 , 6 alcohol wipes 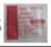 , and 6 bandages 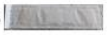

Read through all instructions before proceeding. See reverse side for schedule to collect your DBS. Use the collection kit that was assigned to you, do NOT use barcoded items from another kit.

Step 1. Get a blood collection card from the kit and open up, being sure to not touch the circles. **Write collection date on card.**

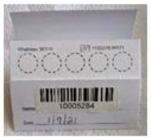

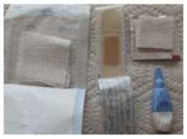

Get a gauze pad, alcohol wipe and bandage from the kit and open up as shown in the picture. Get a lancet from the kit and place with other items on a clean, flat work area.

Step 2. Wash your hands using **warm** water and soap. Dry thoroughly with a clean towel. **This step is very important because rubbing your hands together and using warm water will help to stimulate blood flow to the hands.**

Step 3. Choose a finger to poke on your non-writing (sub-dominant) hand. Use the **side** of the middle or ring finger near the tip. See arrows in the below picture.

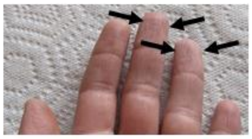

Step 4. Twist cap off lancet and place lancet on work surface.

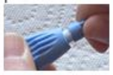

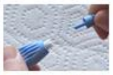

Step 5. Rub hands together for 20 seconds to increase blood flow. Then wipe finger tip with alcohol wipe and allow to air dry.

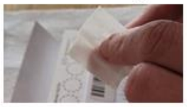

Step 6. Press the lancet firmly to the side of your fingertip until you hear a click and feel a slight pinch.

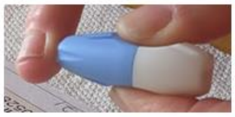

Step 7. Gently massage finger from base to tip to form drop of blood. Wipe away the first drop with a gauze pad.

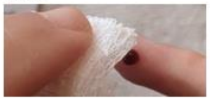

Step 8. Massage finger from base to tip to allow a large blood drop to form.

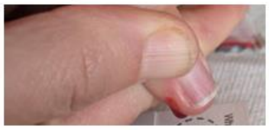

Step 9. Hold finger over the card and allow drop to fall freely onto circle.

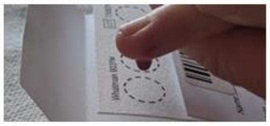

DO NOT touch your finger to the card.
Allow only one drop to fall onto a circle. As the next drop forms, move your finger to the next circle. DO NOT layer drops.
Repeat until you have filled all circles.

Step 10. Use gauze pad to wipe blood from finger, then apply bandage to finger.

Step 11. Let your collection card sit for **at least 3 hours** to dry. Keep card out of direct sunlight. Do not use fans. Blood spots will darken as they dry.

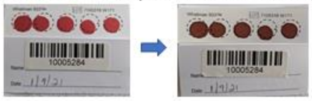

Step 12. Fold flap over blood circles and tuck in. **Complete study questionnaire.** Place card and questionnaire into envelope. Mail envelope using the postal service or deliver in person.

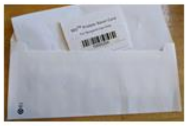

Page 1 of 2 V-021721

Frequently Asked Questions

1. When should I collect my DBS specimens?

If you have not received any doses of the COVID-19 vaccine when first receiving this kit, use the 'Not vaccinated at enrollment' schedule. Otherwise use the 'Vaccinated prior to enrollment' schedule. Based on the selected schedule, write in the anticipated dates of DBS collections in the space provided.

DBS #	Not vaccinated at enrollment	Vaccinated prior to enrollment (one or both doses)	Dates of DBS Collection
1	1-7 days **before** 1st dose of vaccine	Upon receiving the kit	
2	3-4 weeks **after** 1st DBS collection OR 1-7 days **before** 2nd dose of vaccine	3-4 weeks **after** 1st DBS collection	
3	3-4 weeks **after** 2nd DBS collection	3-4 weeks **after** 2nd DBS collection	

2. How do I know if my blood spots are big enough?

Try your best to fill all five circles. However, two completely filled spots will be enough to perform most testing.

Example of a good collection.

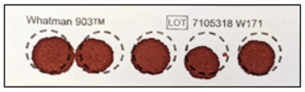

In a good sample, the blood will soak through to the back of the card

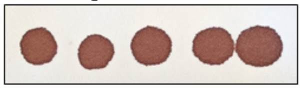

Example of a bad collection. There are only two small spots.

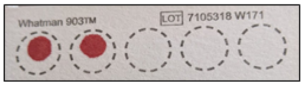

Example of a bad collection. Multiple drops were layered in one circle. Only two spots are partially filled.

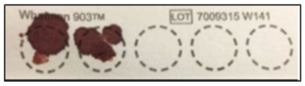

3. I lost my collection kit, what should I do?

Contact us to get a replacement kit. **DO NOT use barcoded items from someone else's kit.** The barcoded ID on your kit was assigned to you and will be used to ensure that you receive the correct results.

4. Is it ok if my blood drops fell outside of the collection circles?

Yes, it is fine if the blood drops don't fit neatly into the circles but try to get them as close as possible.

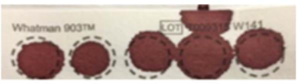

5. I poked my finger but am not getting enough blood out. What should I do?

Try standing up and massaging your finger from base to tip below your waist. You can also try shaking your hand below your waist.

If blood still isn't flowing, it has probably clotted. Wipe your finger with the alcohol wipe and apply a bandage. Try again later, using the extra collection supplies in your kit.

It may help to take a break and move around. This will help stimulate blood flow to your fingers. Then, try again. This time poke another finger or a different spot on the same finger and try the process again.

6. Is it OK to let the blood spots dry overnight?

Yes, the blood should dry completely in 3 hours but it is fine to let the blood dry overnight before mailing.

7. I've changed my mind about participating in this study. What should I do?

You can withdraw from this study at any time for any reason by emailing or calling.

If you have any questions, please contact us.
Thank you for participating in this study!

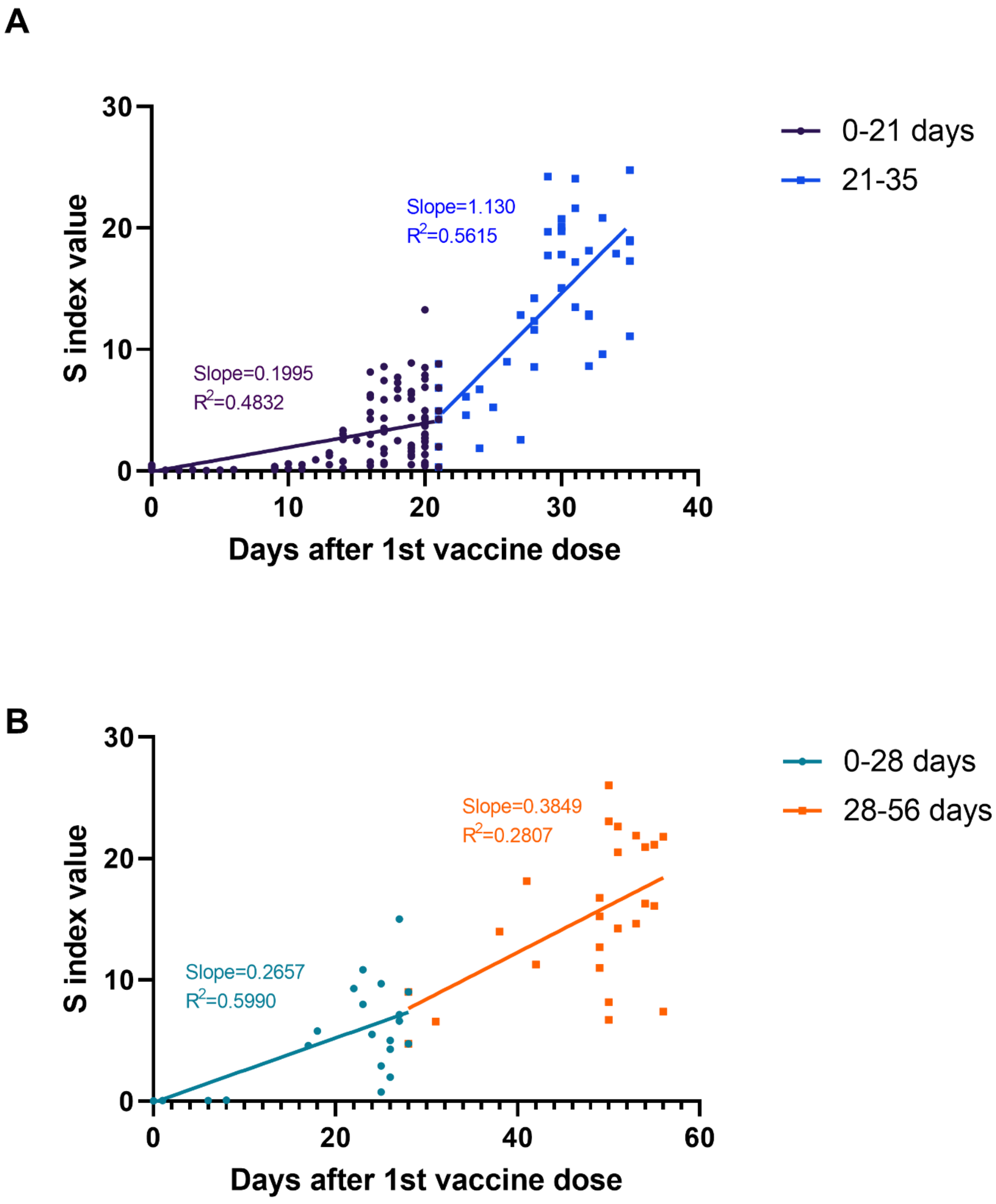

Supplemental Figure 2: Linear regression of naïve individuals who received the (A) Pfizer-BioNTech vaccine or (B) Moderna vaccine from the first dose to the week of peak antibody levels for each group
